# Infusion of* Hibiscus sabdariffa L.* Modulates Oxidative Stress in Patients with Marfan Syndrome

**DOI:** 10.1155/2016/8625203

**Published:** 2016-06-16

**Authors:** María Elena Soto, Alejandra Zuñiga-Muñoz, Verónica Guarner Lans, Erendira Janet Duran-Hernández, Israel Pérez-Torres

**Affiliations:** ^1^Department of Immunology, Instituto Nacional de Cardiología “Ignacio Chávez”, Juan Badiano 1, Sección XVI, Tlalpan, 14080 México City, DF, Mexico; ^2^Department of Pathology, Instituto Nacional de Cardiología “Ignacio Chávez”, Juan Badiano 1, Sección XVI, Tlalpan, 14080 México City, DF, Mexico; ^3^Department of Physiology, Instituto Nacional de Cardiología “Ignacio Chávez”, Juan Badiano 1, Sección XVI, Tlalpan, 14080 México City, DF, Mexico

## Abstract

Marfan syndrome (MFS) is associated with progressive aortic dilatation, endothelial dysfunction, and oxidative stress that contribute to the early acute dissection of the vessel and can end up in rupture of the aorta and sudden death. Many studies have described that the organic acids from* Hibiscus sabdariffa* Linne (HSL) calyces increase cellular antioxidant capacity and decrease oxidative stress. Here we evaluate if the antioxidant properties of HSL infusion improve oxidative stress in MFS patients. Activities of extra cellular super oxide dismutase (ECSOD), glutathione peroxidase (GPx), glutathione-S-transferase (GST), glutathione reductase (GSSG-R), glutathione (GSH), lipid peroxidation (LPO) index, total antioxidant capacity (TAC), and ascorbic acid were determined in plasma from MFS patients. Values before and after 3 months of the treatment with 2% HSL infusion were compared in control and MFS subjects. After treatment, there was a significant decrease in ECSOD (*p* = 0.03), EGPx (*p* = 0.04), GST (*p* = 0.03), GSH (*p* = 0.01), and TAC and ascorbic acid (*p* = 0.02) but GSSG-R activity (*p* = 0.04) and LPO (*p* = 0.02) were increased in MFS patients in comparison to patients receiving the HSL treatment and C subjects. Therefore, the infusion of HSL calyces has antioxidant properties that allow an increase in antioxidant capacity of both the enzymatic and nonenzymatic systems, in the plasma of the MSF patients.

## 1. Introduction

Marfan syndrome (MFS) is an autosomal dominant hereditable disorder of connective tissue caused by the mutation of the gene encoding for tissue fibrillin-1 with highly variable clinical manifestations [1]. This mutation has been associated with alterations in the connective tissue from the eyes, skeleton, and cardiovascular system [2]. Alterations in the cardiovascular system in MFS lead to a variety of pathological conditions such as cardiac arrhythmias, coronary artery disease, left ventricular hypertrophy, congestive heart failure, aortic dilation, aortic dissection, chronic inflammation, endothelial dysfunction, and oxidative stress [3]. Endothelial dysfunction increases the inducible nitric oxide synthase (iNOS) pathway leading to an excess in nitric oxide (NO) production that causes tissue damage. This is associated with generation of peroxynitrite (ONOO^−^) [4]. Similarly, in a MFS mouse model an association of the progression of thoracic aortic aneurysms with endothelial dysfunction caused by reduced glutathione (GSH) depletion has been reported [5]. GSH depletion is the result of reactive oxygen species (ROS) accumulation which favors the vasomotor dysfunction and the cystic necrosis of the aortic wall which is associated with accumulation of oxidative stress in MFS patients [6]. However, the inhibition of ROS production attenuates aneurysm formation in MFS murine model [7]. In addition, other studies in animal models of MFS have shown that the expressions of the precursor enzymes of O_2_^−^, xanthine oxidase, and NADPH oxidase are increased [8]. A previous study from our laboratory has shown an increase in oxidative stress and an alteration of the antioxidant enzymes super oxide dismutase (SOD) and glutathione peroxidase (GPx) in the aortic aneurysm of MFS patients [9]. In addition, antioxidants enzymes such as glutathione peroxidase (GPx) and glutathione-S-transferase (GST) require GSH as cofactor for ROS detoxification [10]. In this reaction GSH is converted to its oxidized form GSSG. Then the reduction of GSSG to GSH is catalyzed by glutathione reductase (GSSG-R) [11]. This GSH system protects from overproduction of ROS under pathological conditions [12].

*Hibiscus sabdariffa* Linne (HSL) is widely cultivated in tropical areas and its calyces are used to prepare hot tea and cold beverages that are consumed worldwide [13]. It is commonly used against hypertension, pyrexia, inflammation, liver disorders, kidney stones, and urinary bladder stones. It is also used as an antibacterial, antifungal, mutagenic agent, as well as a hypocholesterolemic, antispasmodic, and cardioprotective agent [14, 15]. The calyces of HSL contain many chemical constituents including polyphenols, flavonoids such as anthocyanins, delphinidin, hibiscetin, quercetin and gossypetin, protocatechuic acid (PCA), alkaloids, L-ascorbic acid, carotenoids, anisaldehyde, galactose, mucopolysaccharides, pectins, polysaccharides, and stearic acid [13, 16]. Anthocyanin, flavonoids, PCA, and L-ascorbic acid have been demonstrated to have antioxidant effect in vitro and in vivo [13, 16, 17]. Therefore, the aim of this study was to investigate the plasma antioxidant effect of the infusion from HSL calyces in patients with MFS.

## 2. Material and Methods

### 2.1. Study Design

This study is a prospective and observational study that was carried out in one cohort.* Study Population*. The study population consisted of patients who met the requisites to be classified with MFS by Ghent's criteria of 1996 [1].

### 2.2. Patients

17 MFS patients that assisted to the aorta clinic of the National Institute of Cardiology Ignacio Chávez and that were analyzed by an expert rheumatologist were included. The aim of the study was explained to them and subsequently an informed consent letter was delivered to each one. The control (C) group consisted of 10 subjects, without valvular damage that were evaluated previously by an expert cardiologist and rheumatologist to verify that they did not have MFS and routine laboratory tests were made to determine acute phase reactants, triglycerides and HDL cholesterol. Additionally, image studies by echocardiography, computerized tomography, or magnetic resonance were done to discard aortic damage additional to valvular damage. None of the MFS patients and control subjects was taking anti-inflammatory drugs or statins. Medications that could interfere with the outcome of the study such as NSAIDs or lipid-lowering drugs were suspended.

The MFS patients were submitted to a diagnostic protocol that included coagulation tests, X-ray, and electrocardiogram. Aspirin, warfarin, clopidogrel, and other antiplatelet or anticoagulant medications were suspended. The research protocol was approved by the Research and Ethics Committee of our institution (institutional protocol number: 14-900). Informed consent of patients and controls was obtained according to the declaration of Helsinki [18]. 5 mL of blood per patient was centrifuged for 20 min at 936 g to 4°C, the red blood cell pellet was discarded, and the plasma was collected in aliquots of 400 *μ*L and stored at −30°C until used.

### 2.3. Infusion

The HSL calyces were acquired in Chilapa de Alvarez (high zone from Guerrero, México). The infusion was prepared as follows: 20 g of the HSL calyces was added to a liter of boiling (95–100°C) drinking water for 10 min and then left to cool. The solution was filtered and stored at 4°C until used. Patients consumed 1 liter/day for 3 months. To determine total anthocyanin content of the infusion, 100 *μ*L was added to 50 mL of buffers (NaC_2_H_3_O_2_, 4 M) at pH 1 and 4.5, respectively, and the absorbance was measured at 520 and 700 nm and compared against a blank cell, filled with distilled H_2_O. The difference in the absorbance was used in calculating the cyanidin-3-glucoside (total monomeric anthocyanin) as described by the method of Lee [19]. Total flavonoid content was determined by the method of Jia [20]; 100 *μ*L of HSL infusion was added to 2175 *μ*L of distilled H_2_O plus 75 *μ*L of 5% NaNO_2_ and incubated for 3 min. Then, 150 *μ*L of 10% AlCl_3_ was added and the solution was incubated for 5 min. 0.5 mL of 1 M NaOH was added to the mixture and it was shaken vigorously in vortex. The absorbance was measured at 510 nm. The calibration curve was obtained using quercetin as standard. Total estimation of vitamin C was determined by the method of Jagota [21]. 100 *μ*L of the HSL infusion was added to 200 *μ*L of Folin-Ciocalteu reagent 0.20 mM. The mixture was shaken vigorously in a vortex for 5 seconds and incubated for 10 min. The absorbance was measured at 760 nm. The calibration curve was obtained using an ascorbic acid standard solution. The 2% HSL infusion contained 91 ± 39 mg/L of cyanidin-3-glucoside, 12.25 ± 0.32 mg/L of quercetin and 1.03 ± 0.02 mM of vitamin C.

### 2.4. Extra Cellular Superoxide Dismutase Activity

ECSOD enzyme activity was determined in plasma by nondenaturing gel electrophoresis and nitro blue tetrazolium staining as described by Pérez-Torres et al. [22]. 25 *μ*L of plasma was applied directly, without boiling, to a nondenaturing 10% polyacrylamide gel. The electrophoresis was carried out at 120 volts for 4 hours. Subsequently, the gel was incubated in a 2.45 mM nitro blue tetrazolium solution for 20 min, then the liquid was discarded, and the gel was incubated in a 28 mM EDTA solution, containing 36 mM potassium phosphate (pH 7.8) and 0.028 mM riboflavin. After 10 min of incubation under dark conditions, the nitro blue tetrazolium stain for O_2_ was viewed by UV light exposure for another 10 min. Riboflavin and TEMED in the presence of UV light and oxygen produce ROS; nitro blue tetrazolium and SOD compete with them. Where SOD is present the gel remains transparent, whereas reduced nitro blue tetrazolium turns it purple-blue. The gels of ECSOD were analyzed by densitometry by the image analyzer Sigma Scan Pro5. ECSOD activity was calculated following the technique described by Pérez-Torres et al. [22].

### 2.5. Glutathione Peroxidase

For EGPx activity, 100 *μ*L of plasma was suspended in 1.6 mL of 50 mM phosphate buffer (pH 7.3), with added 0.2 mM NADPH, 1 mM GSH, and 1 UI/mL glutathione reductase. The mixture was incubated for 3 minutes at 37°C, then 100 *μ*L of 0.25 mM H_2_O_2_ was added to start the reaction, and the absorbance was monitored for 10 min at 340 nm [23]. Activity is expressed in *μ*mol of NADPH oxidized/min/mL plasma.

### 2.6. Glutathione-S-Transferase

The activity of GST was determined spectrophotometrically. 700 *μ*L of phosphate buffer (0.1 M, pH 6.5) supplemented with 100 *μ*L GSH 0.1 mM and 100 *μ*L 1-chloro-2,4-dinitrobenzene (CDNB) 0.1 mM was added to 100 *μ*L of plasma. The sample was incubated and monitored for 10 min at 37°C at 340 nm. Values of GST activity were expressed in U/min/mL of plasma. A unit of activity of GST is expressed in *μ*mol of GS-DNB conjugate formed/min/mL plasma at 37°C [24].

### 2.7. Glutathione Reductase

To evaluate GSSG-R activity, 700 *μ*L of phosphate buffer 0.2 mM plus 0.5 mM of EDTA pH 7.3, 100 *μ*L of NADPH 0.1 mM, and 100 *μ*L of GSSG 1 mM was added to 100 *μ*L of plasma. It was then incubated and monitored for 10 min at 37°C and the absorbance was read at 340 nm. GSSG-R activity is expressed in U/min/mL of plasma [12].

### 2.8. GSH Concentration

To determinate GSH concentration, 100 *μ*L of plasma previously deproteinized with 20% trichloroacetic acid (vol/vol) and centrifugated to 5000 rpm for 5 minutes was added to 800 *μ*L of phosphate buffer 50 mM, pH 7.3, plus 100 *μ*L of Ellman reactive (5,5′-dithiobis 2-nitrobenzoic) 1 M. The mixture was incubated at room temperature for 5 minutes and absorbance was read at 412 nm. The calibration curve was done with GSH at concentrations from 5 to 25 *μ*mol/mL of plasma [25].

### 2.9. Lipid Peroxidation

50 *μ*L CH_3_-OH with 4% BHT plus phosphate buffer pH 7.4 was added to 100 *μ*L of plasma. The mixture was shaken vigorously in vortex for 5 seconds and then incubated in water bath at 37°C for 30 min. 1.5 mL of 0.8 M thiobarbituric acid was then added and the sample was incubated in a water bath at boiling temperature for 1 hour. After this time and to stop the reaction, the samples were placed on ice; 1 mL 5% KCl was added to each sample as well as 4 mL n-butanol; they were shaken in vortex for 30 seconds and centrifuged at 4000 rpm at room temperature for 2 min. Then the n-butanol phase was extracted and the absorbance was measured at 532 nm. The calibration curve was obtained using tetraethoxypropane as standard [22].

### 2.10. Evaluation of Total Antioxidant Capacity

100 *μ*L of plasma was suspended in 1.5 mL of a reaction mixture prepared as follows: 300 mM acetate buffer pH 3.6, 20 mM hexahydrate of ferric chloride, and 10 mM of 2,4,6-Tris-2-pyridil-s-triazine dissolved in 40 mM chlorhydric acid were added in a relation of 10 : 1 : 1 v/v, respectively. The mixture was shaken vigorously in a vortex for 5 seconds. It was then incubated at 37°C for 15 min in the dark. The absorbance was measured at 593 nm. The calibration curve was obtained using Trolox [26].

### 2.11. Vitamin C

20% trichloroacetic acid was added to 100 *μ*L of plasma. After vigorous shaking the samples were kept in an ice bath for 5 min and centrifuged at 5000 rpm for 5 min; 200 *μ*L of Folin-Ciocalteu reagent 0.20 mM was added to the supernatant. The mixture was shaken vigorously in a vortex for 5 seconds and incubated for 10 min. The absorbance was measured at 760 nm. The calibration curve was obtained using ascorbic acid standard solution [21].

### 2.12. Statistical Analysis

For the analysis of continuous quantitative variables of normal distribution, Student's *t*-test was used. For nonparametric data Mann-Whitney *U* test was employed. The program Sigma Plot version 11, Jandel Corporation, was used to obtain the graphics. The data are presented as mean ± standard error. The differences were considered as statistically significant when *P* < 0.05.

## 3. Results and Discussion

ROS and oxidative stress have been involved in cardiovascular diseases such as arrhythmias, coronary arterial disease, left ventricular hypertrophy, aortic dilatation, aortic dissection, and congestive heart failure [27]. ROS are produced in these diseases through different pathways such as mitochondrial xanthine oxidase, NAD(P)H oxidase, and endothelial nitric oxide synthase decoupling [28]. The role of ROS and oxidative stress in the progression of aortic pathologies such as MFS has been described [5]. MFS is characterized by alteration of vascular function and endothelial dysfunction which promote an increase in the generation of O_2_^−^ leading to an enhanced NO inactivation and increase ONOO^−^. As a consequence, this leads to pseudoaneurysm or aneurysm formation and to obstruction or destruction of the vessel [29].

Different plants have the capacity of reducing the risk of chronic diseases such as hyperlipidemia, hypertension, and cardiovascular disease [30]. Consumption of anthocyanins, polyphenols, and organic acids that are present in HSL has pharmacological effects such as reduction of the risk of coronary heart disease and prevention of some chronic diseases such as atherosclerosis [31]. The benefic physiological effects of these HSL pigments could be related to their potent antioxidant activity which has been demonstrated in various in vitro and in vivo studies [32]. Likewise, PCA, catechin, and (−)-epigallo catechin gallate, also identified in HSL, could act by scavenging harmful free radicals and regenerating other antioxidants to prevent cellular oxidative damages [33]. Therefore, the aim of this study was to investigate the plasma antioxidant effect of the infusion from HSL calyces in patients with MFS.

### 3.1. General Characteristics

The demographical general characteristics and the clinical data of the patients are shown in Table 1. Measurements were made individually and were then grouped to be analyzed; of the total patients 12 were men (67%) and 5 women (33%). The relation of male/female was 2 : 1. The median age of the general group was 23 ± 18 years. The evolution time of disease was 4 years with min and max time (2–20). All subjects met > 2 Ghent criteria. A total of 13 (76.5%) with family history of Marfan disease and all patients had more 7/20 point of the systemic score. The average aortic diameter of the whole group was with median of 55 ± 19 mm. Other characteristics were hypertelorism, bifid uvula, millia, and arterial tortuosity.

### 3.2. Extra Cellular Super Oxide Dismutase

**Figure 1:**
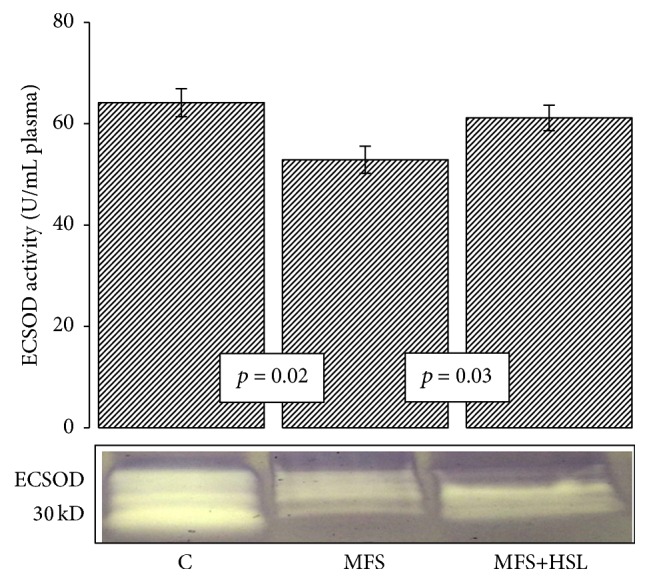
Comparison of the ECSOD activity between experimental groups. C = control, MFS = Marfan syndrome, and MFS+HSL = Marfan syndrome plus* Hibiscus sabdariffa* L. infusion. The lower panel is a native gel representative of the ECSOD activity. The whole scanning shown represents the activity of the enzyme.

The ECSOD is a tetrameric and Zn-Cu glycoprotein with a subunit molecular mass of 30 kDa; it is the main antioxidant enzyme present in extracellular fluids such as plasma, lymph, and synovial fluid [34]. ECSOD is expressed in blood vessels primarily on the surface of vascular smooth muscle cells and the subendothelial space. It contains a binding domain that links it to proteoglycans expressed on cell surfaces. Basal plasma contains 5–20 U/mL^−1^ of ECSOD [35]. ECSOD is an antioxidant enzyme that can protect cells from the potentially harmful effects of ROS. Assays of the activities of this enzyme form part of the indirect determination of the activity of ROS [36]. ECSOD expression can be altered in response to a variety of stimuli including hypertension, atherosclerosis, diabetes, homocysteine, and genetic factors such as polymorphisms in the heparin binding domain [37]. Measurements of ECSOD release to plasma in response to heparin are commonly used as an index of vascular bound ECSOD [38]. The results in this paper show that ECSOD activity is significantly increased in C subjects and MFS+HSL patients (*p* = 0.02 and *p* = 0.03, resp.) in comparison to MFS patients (Figure 1). This result suggests that the loss of ECSOD activity increases oxidative stress in MFS patients. Furthermore, the reduced ECSOD activity in MFS patients could be caused by a background genetic mutation that would also have effects on the vascular function and contribute to the formation of aneurysm [39]. HSL treatment also increases the activity of ECSOD, thus contributing to reduced oxidative stress in MFS patients. A previous study that supports the above-mentioned results showed that inhibition of ECSOD increased vascular oxidative stress and altered the endothelium-dependent vasoreactivity [40]. There is an increased vascular permeability and ischemia in hypertrophic cerebral arterioles in mice with Cu-Zn-SOD deficiency [41]. Others studies in mice lacking ECSOD have suggested that deficiency of this enzyme can lead to increased amounts of free radicals altering of the NO metabolism modifying vascular reactivity [42]. Likewise, it has been described that 12% of the ECSOD functions are related to the activity of some of the GPx isoforms. A decrease of activity of these enzymes results in the accumulation of LPO, increasing oxidative stress [43].

### 3.3. Glutathione Peroxidase

**Figure 2:**
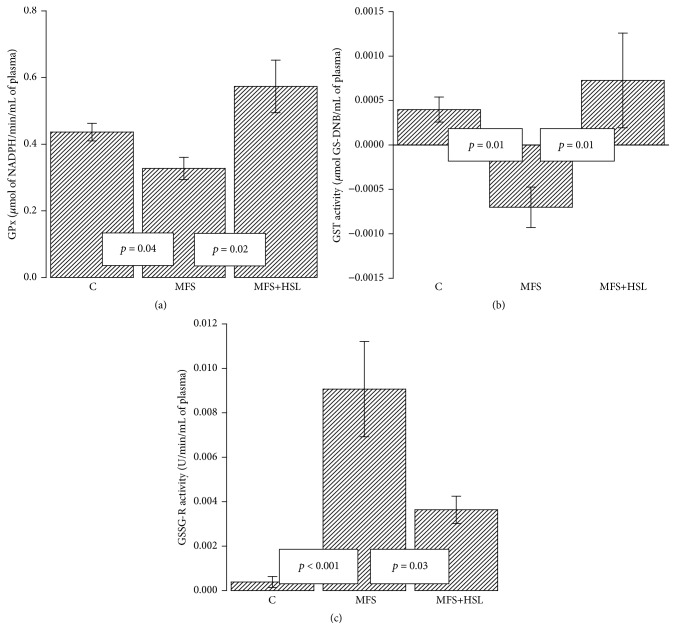
Glutathione peroxidase activity (a), glutathione-S-transferase activity (b), and glutathione reductase activity (c). The three activities of the glutathione employing enzymes were calculated in plasma from experimental groups. C = control, MFS = Marfan syndrome, and MFS+HSL = Marfan syndrome plus* Hibiscus sabdariffa* L. infusion.

Plasma GPx was originally described as a distinct enzyme from cytosolic GPx based on enzymatic and immunological properties and is known as extracellular GPx (EGPx) [44]. EGPx is a unique selenium glycoprotein that reduces organic hydroperoxides, phospholipid hydroperoxides, and hydrogen peroxide in vitro. EGPx is synthesized and secreted predominantly by the kidney [45]. The basal plasma level of this enzyme is of 5–20 U/mL^−1^ [35]; EGPx functions as a major extracellular antioxidant enzyme and participates in the control of ROS induced oxidative stress in the circulation. This enzyme plays a pivotal role in the defense mechanisms of the body against oxidative damage [44]. EGPx metabolized peroxidized organic molecules and H_2_O_2_, and it recycles some of the molecules attacked by H_2_O_2_ having a high affinity and it catalyzes the inactivation of these molecules even at the normal physiological concentrations [45, 46]. EGPx activity represents the initial protective response required to adjust H_2_O_2_ concentration under normal physiological conditions and after and oxidative insult [45]. EGPx activity is reduced by prooxidative conditions such as inflammations and increased concentrations of TNF-*α*, which may induce further ROS accumulation in the circulation [47]. HSL calyces contain anthocyanins that have anti-inflammatory activity and the capacity to downregulate TNF-*α*, thus promoting ROS production [48]. The infusion of HSL might possibly act through its anti-inflammatory effects that decrease TNF-*α* thus lowering ROS concentration. Moreover, several studies in human hypertension and cardiovascular disease have described increased oxidative stress and reduced GPx isoforms expression [46]; therefore, the activity of this enzyme is essential for maintaining the normal vascular function [45]. In our results the activity of EGPx was significantly decreased in the MFS patients compared to C subjects and MFS+HSL patients (*p* = 0.04; *p* = 0.02, resp., Figure 2(a)). This result suggests that the antioxidants properties of HSL may decrease the chronic oxidative stress in MFS patients by favoring the increase in the EGPx activity [47, 48]. A previous study showed that the HSL extract significantly increased GPx and SOD in* Cyprinus carpio* hepatocytes by carbon tetrachloride toxicity [49]. A protective extracellular antioxidant activity against both renal ischemia-reperfusion injury and acetaminophen toxicity was also found in mice that overexpress EGPx [47]. EGPx activity can be inactivated by selenium deficiency or in conditions of oxidative stress in which O_2_^−^ can inhibit the peroxidative function of the enzyme [50]. Symptoms accompanying selenium deficiency in humans and animals demonstrate that it is an essential micronutrient [49]. Selenium might also be decreased in MFS patients as has been described in other cardiovascular pathologies [51]. Selenium participates in the regulation of EGPx, since it is inserted in its active site. Thus, a decrease in selenium levels or its absence can affect the expression and activity of this enzyme [52].

### 3.4. Glutathione-S-Transferase

Another enzyme that participates in the detoxification of ROS is GST. This enzyme catalyzes the glutathionylation of *α*-, *β*-unsaturated aldehydes to produce a conjugation product that is transported from the cell [53]. Its plasma basal level is 0.005 U/mL^−1^ [35]. Our results show that the activity of GST was significantly decreased in the MFS patients when compared to C subjects and MFS+HSL patients (*p* = 0.01, Figure 2(b)). Several pathologies such as hypertension, Takayasu syndrome, and MFS are associated with decreased activity of many antioxidant enzymes including GST and GPx-3 [9]. Our result suggests that the decreased GST activity in MFS patients favors oxidative stress and LPO products and that the antioxidant properties of HSL may contribute to diminishing ROS and the product of LPO which are eliminated by GST and contribute to increase of the GST activity. Therefore, the development of aneurysms and oxidative stress in MFS could be related to the accumulation of the end products of LPO, including 4-hydroxy-2-trans-nonenal (4-HNE) caused by the decreased activity of GST. This finding is consistent with another study where GST activity in the aortic homogenate of MFS patients was decreased [9]. In addition, another study showed a decrease in GST activity related to an increase of ROS in hypertension [54]. It has also been reported that 60% of 4-HNE is metabolized by the GST in vascular cells through conjugation with GSH [53, 55]. This product of LPO, at high concentrations, favors apoptosis in endothelial cells and activates metalloproteinases (MMPs) 1 and 2 in vascular smooth muscle cells. It therefore promotes collagen and elastin degradation in the extracellular matrix [55]. The MMP-2 is associated with the development of inflammation and aneurysms in the thoracic aorta in MFS [56]. However, the HSL calyces contain PCA that can lead to a reduction of matrix metalloproteinase [57]. In GSTA4-null mice, increased levels of 4-HNE also reduced antioxidant capacity and increased apoptosis have been found [53]. Furthermore, the reduction of GST and EGPx activities could be caused by GSH depletion, since both enzymes are GSH-dependents [54]. Likewise, GST catalyzes the nucleophilic attack on no-polar compounds that contain an electrophilic carbon, nitrogen, or sulphur atom by reduced GSH [58]. GST is part of a phase 2 detoxification and catalyzes deactivation of many harmful substances. It also requires GSH as a cofactor for these reactions [53]. Many metabolic pathways result in decrease in GSH and an increase in GSSG concentrations; therefore, the restoration of GSH is crucial for the glutathione redox metabolism [53, 54].

### 3.5. Glutathione Reductase

GSSG-R is the enzyme that restores GSH from oxidized disulfide GSSG form the increases in the enzyme activity positively corresponded to the GSH level [59]. The basal plasma level is of about 0.03 U/mL^−1^ of this enzyme [35]. Our results show that the enzymatic activity of GSSG-R was significantly increased in the MFS patients when compared to the C subjects and MFS+HSL patients (*p* < 0.001 and *p* = 0.03, resp., Figure 2(c)). These results suggest that the increased GSSG-R activity in MFS patients is insufficient to restore the GSH levels that are modified by a chronic oxidative stress in these patients. The HSL treatment decreases the GSSG-R activity which together with the synergistic increases in GST and EGPx and a possible increase of GSH by the HSL treatment could contribute to decrease of oxidative stress [60]. This is probably reflected in the restored amount of GSH in patients treated with the infusion of HSL. In addition, a previous study showed that the polyphenol extract of HSL decreases oxidative stress and increases GSH in liver damage and oxidative stress caused by acetaminophen [61]. Furthermore, prolonged exposure to ROS can exceed the enzyme ability to reduce GSSG to GSH [11].

### 3.6. Glutathione

**Figure 3:**
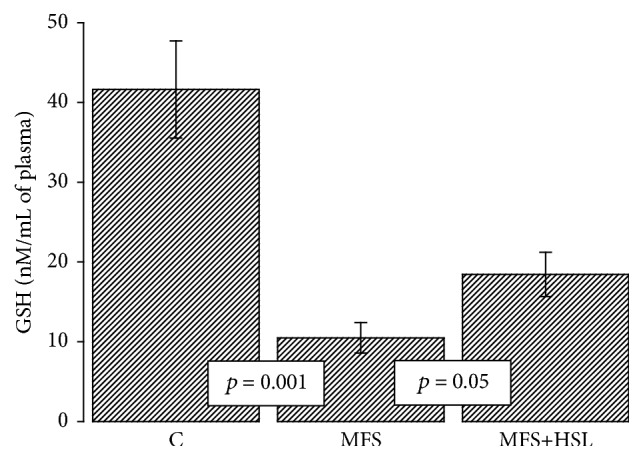
Plasmatic glutathione concentration in experimental groups. C = control, MFS = Marfan syndrome, and MFS+HSL = Marfan syndrome plus* Hibiscus sabdariffa* L. infusion.

GSH is a low molecular weight tripeptide [54]. GSH is the most abundant endogenous intracellular antioxidant present within cells. Approximately 85% of it is in a free form and the rest is bound to proteins [54]. As an antioxidant, GSH has the ability to inactivate O_2_^−^ and OH^−^ radicals and it regenerates vitamins E and C, transforming them into their active forms [62]. GSH plays a central role in the antioxidant defenses and irreversible cell damage happens when the cell is unable to maintain its intracellular concentration [63]. Many investigations have reported that reduced cellular and plasma levels of GSH are an indicator of oxidative stress [64]. Our results show that GSH concentration was significantly diminished in the MFS patients compared to C subjects and MFS+HSL patients (*p* = 0.001 and *p* = 0.05, resp., Figure 3). This result suggests that chronic oxidative stress in MFS patients decreases the plasmatic concentration of GSH but that treatment with HSL can contribute to increase of its concentration, favoring the reduction of oxidative stress in these patients. A deficiency of GSH precursor molecules such as cysteine, glutamate, and glycine or a decrease in the activity of the enzymes that synthesize it such as *γ*-glutamyl-cysteine synthetase and GSH synthetase could be the cause of GSH deficiency in MFS patients [63]. An alteration in the activity of the enzymes that synthesize GSH can predispose not only to GSH deficiency, but also to oxidative stress in these patients.

GSH is an essential factor for the enzymatic function of EGPx and GST and it is also an antioxidant scavenging ROS [64]. The treatment with HSL in MFS patients may contribute to increase of GSH concentration. The PCA present in the calyces of HSL could attenuate oxidative stress complications via elevation of GSH [60]. Furthermore, PCA possesses an inhibitory potential, suppressing the expression of the inducible nitric oxide synthase (iNOS) which is responsible for the NO overproduction that induces oxidative stress. It may also regulate the expression of proinflammatory genes [15]. Is has been described that the expression of iNOS is increased MFS patients, as well as the concentration of nitrates and nitrites in their aortic tissue [9]. Our group recently demonstrated that in the aortic aneurysm of the MFS patients there is an increase in oleic acid which was associated with decreased eNOS but increased iNOS expression and activity. An increase in TNF-*α* and TGF-*β*1, which promotes chronic inflammation and oxidative stress, was also found in these patients [65] The level of iNOS might reflect the degree of inflammation and can be used to evaluate the effects of drugs or alternative products on the inflammatory process [66]. This also indicates that the oxidative damage in these individuals is caused by reactive nitrogen species [9]. When the antioxidant defenses are low, they promote the formation of ONOO^−^. The accumulation of ONOO^−^ within the cell increases in turn cellular oxidative damage [67]. GSH is capable of reducing ONOO^−^ [10]. Another study showed that (−)-epigallo catechin gallate present in de HSL calyces attenuates the severity of oxidative stress and inflammatory response in CCl_4_ induced chronic liver injury involving the downregulation of fibrogenic markers such as TNF-*α*, COX-2, iNOS, and TGF*β*-1 [68]. In addition, TGF-*β*1 directly impairs vasoconstriction, and its overexpression is associated with upregulation of matrix MMPs that cause vascular dysfunction and contribute to aneurysm development in MFS patients [69].

### 3.7. Ascorbic Acid

**Figure 4:**
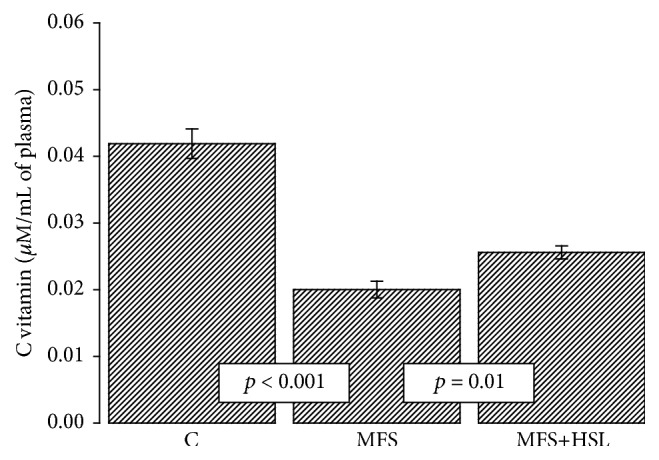
Plasmatic ascorbic acid concentration in experimental groups. C = control, MFS = Marfan syndrome, and MFS+HSL = Marfan syndrome plus* Hibiscus sabdariffa* L. infusion.

Plasma contains an array of antioxidants molecules such as GSH and ascorbic acid whose concentration decreases in the presence of oxidative background [70]. Ascorbic acid is a water-soluble vitamin with a potent antioxidative effect in vivo. Its basal concentration in plasma falls within the *μ*M range [71]. In the present study we found that the plasma ascorbic acid concentration was lowered in MFS patients in comparison with C subjects (*p* < 0.001) and that the HSL treatment restored its concentration (*p* = 0.01, Figure 4). This result suggests that plasma ascorbic acid is decreased by oxidative stress background in MFS patients and that the HSL treatment can restore its concentration [72]. The calyces of HSL are rich in organic acids such as citric and ascorbic acids [72]. In addition, LPO can be inhibited nonenzymatically by GSH and vitamin C. The hydrophilic antioxidants that are present in HSL have scavenging properties by which they are able to inhibit the free radical mechanism of LPO [73].

### 3.8. Lipid Peroxidation and Total Antioxidant Capacity

**Figure 5:**
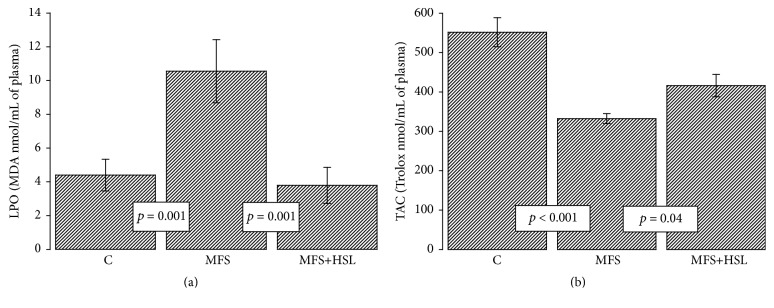
Lipid peroxidation (a) and total antioxidant capacity (b) in experimental groups. LPO and TAC are markers of the nonenzymatic antioxidant system and suggest oxidative stress increase. C = control, MFS = Marfan syndrome, and MFS+HSL = Marfan syndrome plus* Hibiscus sabdariffa* L. infusion.

Several oxygen species such as H_2_O_2_ can be degraded to HO^−^ by the Fenton reaction, producing high levels of LPO [74]. Lipids are a main target of oxidative attack and this leads to formation and accumulation of LPO products [75]. LPO product accumulation in human tissues is a major cause of tissue and cell dysfunction that plays a major role in oxidative stress related diseases [76, 77]. In this study LPO level in plasma of MFS was significantly elevated in comparison to C subjects and treatment with the HSL infusion significantly suppressed it (*p* = 0.001, Figure 5(a)). The level of LPO in cells is controlled by various cellular defense mechanisms consisting of enzymatic and nonenzymatic scavenger systems [77]. These enzymatic systems are altered in MFS patients as described in this paper. The treatment with HSL decreased this condition. The results of this study showed that the TAC, a marker the nonenzymatic antioxidant system, was significantly decreased in the MFS patients compared with the control subjects and MFS+HSL patients (*p* < 0.001 and *p* = 0.04, resp., Figure 5(b)). In vivo and in normal conditions, there is an abundance of numerous small molecules such as GHS and vitamins increasing the nonenzymatic antioxidant system; however, under chronic oxidative stress these reserves can be depleted [78]. The results of the measurement of LPO and TAC suggest that the low GSH level in MFS patients may be related to the apparent increase in LPO in plasma and that the treatment with HSL may contribute to decrease of LPO. This condition favors an increase in the nonenzymatic antioxidant system, across of the increase from GSH concentration [77].

GSH has the capacity to trap HO^−^ and O_2_^−^, and it regenerates vitamins E and C to their active form [13, 78]. Thus the increase in the concentration of GSH could partly explain the lowering of LPO and the elevation of the antioxidant capacity of the nonenzymatic system observed in the MFS patients treated with HSL. LPO products can readily react with GSH; this reaction can occur spontaneously but is several hundred times faster when it is catalyzed by GST. The conjugation of LPO products with GSH is considered as a detoxification step, facilitating its urinary excretion [58]. In addition, many studies have shown that the treatment with HSL decreases the LPO. For example, an HSL infusion in streptozotocin diabetic rats increased the activity of catalase and GSH and reduced LPO [16]. The HSL extracted with chloroform and ethyl acetate significantly inhibited the formation of LPO induced by t-BHT in rat primary hepatocyte cultures [77]; HSL aqueous extracts have also been found to decrease LPO concentration and LDL oxidation. This effect was attributed to the metabolite PCA or to the native form cyanidin-3-glucoside [17]. This is supported by the fact that the cyanidin-3-glucoside can react with peroxyl radicals and be converted in vitro to PCA. This suggests that cyanidin-3-glucoside would induce the production of another radical scavenger that might react with free radicals [70]. Studies in vitro have shown that the polyphenolic fraction from aqueous HSL extracts increases the TAC [61]. The HSL polyphenols may participate as captors of ROS in a second line of defense when they have not been neutralized by the enzymatic antioxidant system [13].

## 4. Conclusions and Perspectives

The MFS patients present chronic oxidative stress that participates in aneurysm formation. The infusion of HSL calyces has antioxidant properties that allow for the increase in antioxidant capacity of both the enzymatic and nonenzymatic systems, in the plasma of the MSF patients. These antioxidant systems are essential for the homeostasis of the redox state and to maintain low oxidative stress. The application of antioxidants and other active therapies could help in the prevention and mitigation of adverse oxidative stress in the MFS patients and thereby beneficially impact on patient survival.

These relevant findings suggest the need of conducting multicentric studies or systematic studies to provide therapies with antioxidants that improve the redox state of these patients and that are appropriate to the clinical context of each particular subject.

## Figures and Tables

**Table 1:** Demographics general and characteristics of the Marfan syndrome patients.

Number	Gender	Age	Time course of the disease	Family history	Lens dislocation	Systemic score	Aortic diameter (mm)	*Z*-score	Dissection	Mutation gen
1	F	58	4	+	−	8/20	68		+	FBN-1 and TGFBR2 negative
2	F	23	6	+	−	10/20	33		No data	FBN-1 and TGFBR2 negative
3	M	12	9	+	+	9/20	32	3.79	No data	FBN-1 positive, 42 exons
4	M	10	6	+	+	10/20	35	4.7	No data	FBN-1 positive, 14 exons
5	M	57	12	+	+	7/20	70		+	FBN-1 positive, 42 and 28 exons
6	M	57	4	+	−	9/20	58		No data	FBN-1 negative and TGFBR2
7	M	23	2	−	+	7/20	91		+	FBN-1 positive, 28 exons
8	M	58	2	−	+	7/20	68		+	—
9	M	28	6	+	−	9/20	70		−	FBN-1 positive, 14 exons
10	M	37	3	−	−	7/20	55		−	FBN-1 positive, 14 exons
11	M	46	20	+	+	7/20	80		−	FBN-1 positive, 42 exons
12	M	32	13	−	−	9/20	38		−	—
13	M	12	4	+	−	7/20	30	3.04	−	TGFBR2 positive, 6 exons
14	F	17	4	+	−	6/20	26	1.05	−	TGFBR2 positive, 6 exons
15	F	16	4	+	−	7/20	49	6.42	−	TGFBR2 positive, 6 exons
16	M	12	4	+	−	9/20	35	4.42	−	FBN-1 and TGFBR2 negative
17	F	22	4	+	−	7/20	57		+	FBN-1 positive, 14 exons and positive TGFBR2, 6 exons

F = female and M = male. *Z*-score was adjusting the measure aortic diameter age and weight.

## Additional Points

This study has as an important limitation of the small size of the MFS patient sample. This pathology occurs with an incidence of 2-3 per 10000 individuals, being autosomal dominant disorders of the connective tissue caused by mutations. Therefore, the follow-up of the experimental project was complex. The controls included were perfectly studied in order to determine that they were the best possible controls.
